# Echec des étudiants du deuxième cycle d´études médicales à la faculté de médecine de Nouakchott: facteurs prédictifs et suggestions de résolution

**DOI:** 10.11604/pamj.2020.37.250.19871

**Published:** 2020-11-18

**Authors:** Mohamed Jiddou Sidi Baba, Ahmedou Moulaye Idriss, Yahya Tfeil

**Affiliations:** 1 Department of Surgical Specialties, Faculty of Medicine, Nouakchott Al Aasriya University, Nouakchott, Mauritania

**Keywords:** Echec scolaire, redoublement, pédagogie, médecine, Nouakchott

## Aux éditeurs of Pan African Medical Journal

En Mauritanie la plupart des centres de santé au niveau périphérique sont tenus et gérés par des non-médecins. Ce déficit est constaté dans toute l'Afrique au sud du Sahara [1]. Pour faire face à cette situation inquiétante, l´effectif des étudiants de la faculté de médecine de Nouakchott a beaucoup accru ces dernières années. Néanmoins, le nombre de médecins sortants reste encore faible. Cet échec est vécu de manière dramatique aussi bien par l´étudiant que par sa famille, mais aussi par les formateurs. Il est en même temps l´échec des institutions universitaires qui demeurent incapables de l´endiguer malgré les moyens alloués par les pouvoirs publics [2]. Les étudiants ayant interrompu leurs études, invoquent le plus souvent pour expliquer leur abandon la perte d´intérêt pour la discipline, une pédagogie inadéquate et le manque d´encadrement de la part des enseignants [3]. L´objectif de ce travail est de déterminer les facteurs prédictifs de l´échec des étudiants du deuxième cycle d´études médicales (DCEM) à la faculté de médecine de Nouakchott et de proposer des solutions appropriées.

Il s'agit d'une étude mixte, observationnelle, transversale à visée descriptive et analytique réalisée à la faculté de médecine de l'Université de Nouakchott. Les données ont été analysées par le logiciel SPSS, v20. Le focus groupe avait concerné dix étudiants qui avaient discuté une cinquante d´idées émises par les 22 étudiants en réponse à la question: « quelles sont à votre avis les améliorations supplémentaires à apporter de manière générale pour empêcher l´échec ? »

Sur 449 étudiants en DCEM, 27 (6%) avaient été inscrits au moins trois fois dans une même classe. Le taux de participation à l´étude était de 81,4% (22/27). Parmi les 22 étudiants qui participaient à la présente étude, 16 (72%) étaient de sexe masculin d´où un sexe ratio de 2,6. L´âge moyen était de 27,73 (22-35), la médiane était de 27 ans et le mode était de 26 ans. La tranche d´âge la plus représentée était 26-30 (68.2%). Il y avait 10 (45,5%) inscrits en DCEM1 ,10 (45,5) en DCEM2, 2 (9,1%) étudiants étaient inscrits en DCEM4 et aucun étudiant de la DCEM3 n´était concerné. Le nombre d´étudiants ayant 2 redoublements dans la même classe était de 14 (63,6%), Quinze étudiants (68,2%) n´avaient pas de problèmes de compréhension du cours, 14 (63,2%) trouvaient que les supports pédagogiques étaient disponibles. Treize (59,1%) étudiants trouvaient qu´il n´existait pas un soutien pédagogique de la part du décanat. Seize (72,7%) étudiants ont signalé ne pas avoir eu la visite d´un responsable de la faculté durant la période de leur stage et 17 (77,3%) stagiaires ont confirmé que la durée du stage était effectuée telle que prévue au départ. Les étudiants ayant une opinion favorable sur le stage représentaient 45,5%. Les réponses à la question: « quelles sont à votre avis les améliorations supplémentaires à apporter de manière générale pour empêcher l´échec ? » Les cinq idées les plus pertinentes retenues par le focus groupe sont par ordre décroissant: un meilleur encadrement au stage, des cours interactifs suivi de travaux pratiques et dirigés pour une meilleure compréhension, l´existence d´un examen d´évaluation des étudiants au stage, la mise en place d´un service chargé des problèmes sociaux des étudiants et un appui financier.

L´âge moyen de notre étude est de 27,73 ans, ce qui est supérieur à l´âge moyen (21 et 23 ans) de l´étude realisée par Meziani *et al*. [4] en 2006 à Paris VI. La prédominance masculine est nette (72%), en adéquation avec le taux 64,4% retrouvé au Benin par Adoukonou *et al*. [5] contrairement à l´étude publiée en 2017 et réalisée en Nouvelle Zélande par Jardine *et al*. [6] qui retrouve un taux 41%. Le taux d´échec dans notre étude est de 6%. Celui-ci reste inférieur au taux retrouvé par la faculté de médecine de Cotonou, soit 20% [5]. Cette différence peut être expliquée par le taux important de l´échec dans le PCEM concerné par cette étude. L´inexistence de travaux pratiques et dirigés n´était pas en mesure de combler le déficit de la compréhension des cours théoriques souvent jugé pléthoriques sans objectifs et mal compris. Les problèmes de santé, la gestion de foyer pour les mariées, le transport, le foyer, le restaurant universitaire et la coupure de la bourse pour des étudiants en échec sont tant de difficultés qui jouent un rôle déterminant dans l´échec de nos étudiants. Ces facteurs non cognitifs (capacité financière, stabilité psychologique et maturité) ont été retrouvés dans l´étude réalisée au Nigeria en 2007 par Afolabi *et al*. [7].

A l´issu de cette étude, nous constatons que les méthodes pédagogiques sont centrées essentiellement sur les cours magistraux qui sont le plus souvent mal compris, alors que les travaux pratiques et dirigés sont quasi-inexistants. Le manque d´un service social au sein de la faculté, l´éloignement de la faculté, la coupure de la bourse et l´inexistence de restaurant et de foyer universitaire sont autant de facteurs qui jouent un rôle déterminant dans l´échec des étudiants. Enfin, l´échec des étudiants de DCEM pourrait être plus inquiétant si on y rajoute celui des étudiants du premier cycle d´étude médicale d´où l´intérêt de faire une étude de plus grande ampleur qui concernera tous les étudiants de la faculté afin de déterminer de façon objective et pertinente les facteurs prédictifs de l´échec des étudiants de la faculté de médecine de Nouakchott et les réponses appropriées.
